# Inhibition of Biofilm Production and Determination of In Vitro Time-Kill *Thymus vulgaris* L. Essential Oil (TEO) for the Control of Mastitis in Small Ruminants

**DOI:** 10.3390/pathogens14050412

**Published:** 2025-04-24

**Authors:** Michela Galgano, Francesco Pellegrini, Daniela Mrenoshki, Luciana Addante, Alessio Sposato, Laura Del Sambro, Loredana Capozzi, Elisabetta Catalano, Marianna Solito, Francesco D’Amico, Davide Messina, Antonio Parisi, Annamaria Pratelli, Paolo Capozza

**Affiliations:** 1 Istituto Zooprofilattico Sperimentale Della Puglia e Della Basilicata, Via Manfredonia 20, 71121 Foggia, Italy; michela.galgano@izspb.it (M.G.); luciana.addante@izspb.it (L.A.); alessio.sposato@izspb.it (A.S.); laura.delsambro@izspb.it (L.D.S.); loredana.capozzi@izspb.it (L.C.); elisabetta.catalano@izspb.it (E.C.); solito.marianna@gmail.com (M.S.); antonio.parisi@izspb.it (A.P.); 2 Department of Veterinary Medicine, University Aldo Moro of Bari, Sp Casamassima Km 3, Valenzano, 70010 Bari, Italy; danimrenoski@yahoo.it (D.M.); annamaria.pratelli@uniba.it (A.P.); paolo.capozza@uniba.it (P.C.); 3 Department of Public Health, Experimental and Forensic Medicine, University of Pavia, via Carlo Forlanini, 27100 Pavia, Italy; 4 Istituto Zooprofilattico Sperimentale del Piemonte, Della Liguria e Della Valle d’Aosta, S.S. Genova e Portualità Marittima, 16129 Genova, Italy; francesco.damico@izsplv.it; 5 Division of Veterinary Clinical Science, School of Veterinary Medicine and Science, Faculty of Medicine and Health Sciences, University of Nottingham, Sutton Bonington Campus, Loughborough LE12 5RD, UK; davide.messina1@nottingham.ac.uk

**Keywords:** mastitis, biofilm, thyme essential oil, *Staphylococcus* spp., time-kill

## Abstract

*Staphylococcus aureus* and coagulase-negative staphylococci (CNS) are the main causative agents of mastitis in sheep. Their ability to form biofilms in vivo is considered an important virulence factor underlying mastitis outbreaks refractory to antibiotic treatments. Furthermore, pre- and postdipping immersion during milking in iodine substances could determine the presence of residues in milk and therefore represent a health risk factor for consumers. The aim of this study was to evaluate the antibacterial and biofilm inhibitory activity of *Thymus vulgaris* L. essential oil (TEO) against staphylococci strains isolated from ovine clinical mastitis. In particular, 3 reference strains (*S. aureus* 25923 and 11623 and *S. epidermidis* 12228) and 12 clinical isolates (6 *S. aureus* and 6 CNS) were used. TEO solutions, from a concentration of 1% (*v*/*v*) to 1.25% (*v*/*v*), corresponding to 9.28–2.32 mg/mL, were obtained after solubilization in 10% dimethyl sulfoxide (DMSO) and used to evaluate the bacterial time-kill compared to that of an iodine-based solution. Antibacterial efficacy was then assessed by the minimum inhibitory concentration (MIC) and minimum bactericidal concentration (MBC), while biofilm inhibition was assessed by minimum biofilm inhibitory concentration (MBIC) using a spectrophotometer at a wavelength of 570 nm. Additionally, biofilm-associated genes (icaA and icaD) were evaluated in all tested strains by PCR. The tested TEO concentrations were able to significantly and prominently reduce bacterial growth compared to controls, as demonstrated by bacterial time-kills. The MIC value was obtained at a concentration of 0.50% (*v*/*v*) for a single coagulation-positive isolate (*S. aureus* (f)) and at a concentration of 0.25% (*v*/*v*) for all other isolates. TEO showed effective bactericidal action with a 99.9% reduction in CFU/mL of all isolates in the MBC test at a concentration of 0.25% (*v*/*v*) for most of the tested strains. Furthermore, a marked inhibition in biofilm formation at all tested concentrations was observed, with MBIC value of 0.25%. All *S. aureus* tested were biofilm-producing strains and positive for icaA and icaD genes, while two CNS biofilm-producing strains were negative for both genes. These preliminary results suggest that TEO could be a promising alternative as an udder disinfectant during milking practices. Although in vivo studies are needed to confirm the efficacy and safety of TEO as an adjuvant in the prevention and treatment of udder infections, TEO could help counteract the emergence of antimicrobial resistance and reduce the potential risk of iodine residues in milk.

## 1. Introduction

Mastitis is an inflammation of the mammary glands usually caused by bacteria that can severely affect the farm economy due to milk production loss, impairment of milk quality, an increased use of drugs resulting in multidrug resistance and/or the presence of antibiotic residues in dairy products, as well as an increase in the number of involuntary cullings [1]. Mastitis can be classified as clinical, subclinical, and chronic based on the degree and progression of the inflammation [2]. In recent years, the incidence of subclinical mastitis has increased, becoming a potential source of contamination of raw milk intended for humans or for the production of raw-milk cheese. Based on these data, it is necessary to emphasize the sanitary aspect of the possible impacts on human health [3].

*S. aureus* and coagulase-negative Staphylococci (CNS) are the most important causative agents of mastitis [4]. The pathogenesis of *Staphylococcus* spp. mastitis is attributed to a combination of management, extracellular factors, and properties such as adherence and biofilm formation [5]. Indeed, some *Staphylococcus* spp. are able to produce biofilm, a complex polysaccharide- or protein-bound bacteria structure that facilitates adhesion and replication on environmental surfaces and on animal tissues [6]. Bacteria in biofilm are able to resist phagocytosis, antimicrobial agents, and disinfectants due to poor diffusion through the matrix, and they can alter cellular metabolism [6]. Molecular studies have shown that microbial biofilm formation is encoded by certain biofilm-associated genes, mainly 12, i.e., fibrinogen-binding proteins (*fib*), fibronectin-binding proteins (*fnbA* and *fnbB*), intercellular adhesion (*ica ABCD*), clumping factor (*clfA* and *clfB*), elastin-binding protein (*EbpS*), laminin-binding protein (*lbp*), and collagen-binding protein (*cna*) genes in *Staphylococcus aureus* biofilm formation [7,8]. Each of these has a role in biofilm formation. However, it is known that the locus of intercellular adhesion of *ica*, composed of a four-gene operon (*ica ABCD*), is the main culprit responsible for encoding the essential proteins for the production of polysaccharide intercellular adhesion (PIA), which mediates the cell–cell adhesion process, thus facilitating the biofilm formation of *Staphylococcus* spp. [9,10,11]. Among *ica* genes, *icaA* and *icaD* were reported to play a significant role in biofilm formation in *Staphylococcus aureus* and in other Staphylococcal species [11,12,13,14,15]. Hence, research attention has turned to the genotyping of genes responsible for biofilms through the detection of *icaA* and *icaD* along with the phenotypic detection of biofilm in *Staphylococcus* spp. isolates in order to develop new strategies for the prevention or effective treatment of mastitis [16].

In dairy farming, the prevention of external udder infections is essential for milking hygiene and is performed by soaking the teat (nipple) in an antiseptic solution, such as an iodine-based solution, or by applying the solution to the external mammary epithelium [17]. Commonly used antiseptics are synthetic chemicals whose residues in milk can compromise consumer health and the quality of dairy products [18,19]. Natural antimicrobial agents, such as propolis, tannin, or essential oils (EOs), are of interest for the creation of synthetic antimicrobial or antiseptics drugs, especially when used in suitable forms and concentrations [20]. EOs contain various chemical constituents, such as monoterpenes, sesquiterpenes, diterpenes, and other aromatic or aliphatic compounds, which possess therapeutic properties including antibacterial, antifungal, and antiviral activities, as well as anti-inflammatory and anti-oxidation effects [21,22]. Recent data showed EOs’ effectiveness against the bacteria responsible for mastitis [20,23] and against biofilm formation and in addition showed that they represent an interesting alternative to conventional drugs since they are safe to use, easy to prepare, and inexpensive, and no resistance has been reported among pathogens responsible for mastitis [24,25]. It should also be emphasized that unlike disinfectants and antibiotics for which bacteria have developed increasingly effective resistance mechanisms, EOs are able to inhibit several bacterial cellular mechanisms, such as peptidoglycan synthesis, as well as to modify membrane hydrophobicity and modulate quorum sensing, being also less susceptible to the development of resistance [26]. In fact, conventional antibiotics are limited by their narrow spectrum of activity due to the specific targeting mechanism often aimed at a single molecular target or pathway, towards which there is an increasing development of resistance among pathogenic microorganisms. In contrast, EOs, due to their complex compositions, are capable of synergistic interactions against different bacterial target sites, enhancing their antimicrobial efficacy and making the development of resistance difficult. Therefore, EOs may represent a promising alternative to antibiotics [27].

Given the increasing consumption of fresh milk and dairy products from species other than cattle, such as goats and sheep, due to their high nutritional properties [28], the aim of this study was to evaluate the antibacterial and antibiofilm action of thyme (*Thymus vulgaris*) essential oil (TEO) at different concentrations against the bacteria responsible for subclinical mastitis isolated from sheep in farms in the province of Bari (Apulia, Italy). Furthermore, to improve the reliability of the results on the ability of TEO to inhibit biofilm, the phenotypic and genetic expressions of *icaA* and *icaD* genes were evaluated, since the phenotype of these genes does not always correlate to the presence of the genes. However their presence may represent an important virulence factor in subclinical mastitis [29,30]. In addition, the activities of iodine and TEO were tested and compared in order to provide useful data to stimulate interest in these natural molecules to eventually replace chemical agents, which may leave residue in dairy products, with possible repercussions on consumer health, especially for those in more sensitive categories [18].

## 2. Materials and Methods

### 2.1. Essential Oils

The pure EO of *Thymus vulgaris* (TEO), provided by Specchiasol S.r.l. (Bussolengo, VR, Italy), was stored in a brown glass bottle at a temperature of 0–4 °C. Solvents (analytical grade), n-alkanes standard mixture C10–C40, and all standard compounds were purchased from Supelco Sigma-Aldrich S.r.l. (Milano, Italy). Filters were supplied by Agilent Technologies Italia S.p.a (Milano, Italy). TEO was selected based on the literature references [22,31]. The percentage values of the components of TEO were detected by the gas chromatography–mass spectrometry (GC/MS) technique [32,33], as reported by Galgano et al. [22].

Briefly, chromatographic analyses of TEO were performed on an Agilent 6890 N gas chromatograph equipped with a 5973 N mass spectrometer, provided with an HP-5 MS (5% phenylmethylpolysiloxane, 30 m, 0.25 mm i.d., 0.1 μm film thickness capillary column). The temperature program was set as follows: 5 min at 60 °C, then 4 °C/min to 220 °C, then 11 °C/min to 280 °C, held for 15 min, for a total run of 65 min. Injector and detector temperatures were 280 °C; the carrier gas was He; the flow rate was 1 mL/min; the split ratio was 1:50; the acquisition range was 29–400 *m*/*z* in electron-impact (EI) mode; and the ionization voltage was 70 eV.

### 2.2. Compounds Identification and Dilution Design

For chemical characterization, TEO was diluted 1:100 (*v*/*v*) in ethyl-acetate and after filtration, 1 µL of each EO solution was injected into the GC-MS. Qualitative analyses were carried out by comparing the calculated linear retention indices (LRIs) and similarity index of mass spectra (SI/MS) for the obtained peaks with the arithmetic index (AI) and the analogous data reported in the literature [33] and in the NIST 2017 databases (NIST 17, 2017. Mass Spectral Library-NIST/EPA/NIH. Gaithersburg, MD, USA: National Institute of Standards and Technology. Last accessed on 12_2021), respectively. The LRI of each compound was determined using temperature programming analysis and was calculated using the Van den Dool and Kratz equation [32] related to a homologous series of n-alkanes (C10–C40) under the same operating conditions. The SI/MS values were determined as reported by Koo et al. [34].

The relative percentages of the components were calculated based on GC peak areas without using correction factors (Table S1).

To obtain a correct solubilization of TEO, suitable for all experimental procedures, the stock solution was serially diluted in 5 mL of Brain Heart Infusion (BHI) (Oxoid, Milan, Italy) with 10% dimethyl sulfoxide (DMSO) (Thermo Fisher, Rome, Italy), resulting in solutions ranging from a concentration of 1% (*v*/*v*) to 1.25% (*v*/*v*), corresponding to 9.28–2.32 mg/mL. The emulsion was then sonicated for 30 min and vortexed for 8 min to obtain a stable oil emulsion, as reported by Galgano et al. [25].

### 2.3. Bacteria Strains

Twelve strains of *Staphylococcus* spp. were selected. The strains were isolated from raw-milk samples of dairy sheep with diagnosed subclinical mastitis in a dairy farm located in south Italy (Apulia) during a project for the evaluation of the efficacy of fluoroquinolone antibiotics. The samples were collected by the farm veterinarian before receiving any antibiotic therapy. Presumptive identification was made by Gram stain, and further confirmation was made based on biochemical tests, such as catalase, coagulase, and Api 20 Sthap (API 20 Staph System, BioMérieux, Marcy-l’Étoile, France). Isolated bacteria, stored at −80 °C, were transferred in 2 mL Brain Heart Infusion broth (BHIb) (Oxoid, Milan, Italy) and incubated at 37 °C for 24 h. Three reference strains, two *S. aureus* and one *S. epidermidis*, were used: one well-known slime-producing strain ATCC 25923, to evaluate biofilm production by the isolates, one well-known MRSA strain ATCC 11623 (Biogenetics Diagnostics, Padova, Italy), to evaluate the antibiotic resistance to penicillin of the isolates/reference strain for antimicrobial resistance research and biofilm production, and one well-known nonslime-producing *S. epidermidis* strain ATCC 12228 (Biogenetics Diagnostics, Italy), as a negative control for biofilm production.

### 2.4. Screening for MDR Activity

Thirteen different antibiotics (Liofilchem, Teramo, Italy), amoxicillin (AML; 30 μg), ampicillin (AMP; 10 μg), penicillin (P; 10 μg), cefalexin (CL; 30 μg), cefoxitin (FOX; 30 μg), ceftriaxone (CRO; 30 μg), clindamycin (CD; 2 µg), tetracycline (TE; 30 μg), oxacillin (OX; 1 μg), gentamicin (CN; 10 μg), erythromycin (E; 15 µg), rifampicin (RD; 30 μg), and enrofloxacin (ENR; 5 μg), were used to investigate in vitro the antimicrobial activity of all isolated *Staphylococcus* strains using the disk diffusion method (DDM). The antibiotics were selected based on EFSA guidelines, the available literature [35], and according to the Clinical & Laboratory Standards Institute (CLSI) guidelines. Penicillin (10 μg), oxacillin (1 µg), and cefoxitin (30 µg) disks were used to identify methicillin resistant strains (MRSA) by disk diffusion method, and two strains resulted *Staphylococcus* aureus methicillin susceptibl (MSSA), as reported by Mimica et al. [36].

The European Committee for Antimicrobial Susceptibility Testing (EUCAST) (http://www.eucast.org/clinical_breakpoints/, accessed on 12_October 2024) guidelines were used for the interpretation of the test after incubation at 37 °C for 24 h. Based on EUCAST imperative criteria, the isolate strains were categorized as susceptible (S) or resistant (R).

### 2.5. Screening for TEO Activity

#### 2.5.1. Disk Diffusion and Broth Microdilution Methods

The disk diffusion method was carried out as previously described [37,38]. The bacterial suspension, prepared in a sterile saline solution, was adjusted to a density of 1.0 × 10^8^ UFC/mL cells. A sterile swab immersed in the bacterial suspension was used to inoculate the entire surface of Muller Hinton agar (MH) (Oxoid, Milan, Italy). One plate was prepared for each oil and 10 µL of each EO dilution (1%, 0.5%, and 0.25%, corresponding to 9.28 mg/mL, 4.64 mg/mL, and 2.32 mg/mL) was applied on a sterile blank disk (Oxoid, Milan, Italy) aseptically placed on the inoculated plates. Then the plates were incubated for 24 h at 37 °C. After incubation, the inhibition zones were measured in millimeters, according to the literature [37,38], with a blank disk diameter of 6 mm.

The broth microdilution method looked for the minimum inhibitory concentration (MIC) and minimum bactericidal concentration (MBC).

Tests for the MIC and MBC of TEO against isolated bacteria were performed in triplicate by broth microdilution method according to the CLSI recommendation (2009) with some modifications. Microdilution broth assays were determined in 96-well microtiter plates (SigmaAldrich, Costar, Saint Louis, MO, USA). Each strain at a density of 0.5 McFarland (10^8^ CFU/mL), calculated with a densitometer (Densichek, BioMérieux, Durham, NC, USA), in 0.85% saline was put in contact with different concentrations of TEO, from 1% to 0.25% (*v*/*v*), in a final volume of 200 µL. Each solution was tested in triplicate. In addition, a negative control (BHI broth with DMSO: PBS) and a positive control (BHI broth with DMSO: PBS and bacterial inoculum without EO) were prepared for each solution tested in every plate. All plates were incubated at 37 °C for 24 h. The MIC was defined as the lowest concentration of TEO that prevented visible growth after 24 h incubation.

Samples of 100 µL from all wells with no visible growth, including negative and positive control, were inoculated on to PCA (Plate Count Agar) (Oxoid, Milan, Italy) and incubated at 37 °C for 24 h. The MBC was considered as the lowest concentration of TEO that showed no growth on PCA.

#### 2.5.2. Determination of Time-Kill Kinetics

TEO concentrations which showed the highest antibacterial activity against bacteria isolated in the broth microdilution test were selected to determine the time-kill kinetics. The test was performed according to the method of Chamdit and Siripermpool [39] with some modification. Briefly, a 100 µL aliquot of bacterial suspension (10^8^ CFU/mL) was added to a tube containing 2 mL BHI supplemented with the selected TEO concentration (0.25% (*v*/*v*)), then mixed on a vortex for 1 min. After 0.5 h, 1 h, 2 h, 3 h, 4 h, 5 h, 6 h, and 24 h of contact at 37 °C, 200 µL of the mixture was 10-fold diluted with 0.89% sodium chloride solution to stop the antimicrobial effect of TEO. An aliquot of 100 µL from 10^−1^ to 10^−6^ dilutions of each tube at each contact time was spread on PCA plates and then incubated at 37 °C for 24 h. The grown colonies were counted and recorded. The same protocol was also performed with Povi Iodine 100-PF081 solution at 0.25% (*v*/*v*) (Nuova Farmec s.r.l.—Groupe ANIOS, Pescantina, Italy), which appears to be the most widely used solution before and after milking to prevent the onset of mastitis [40]. This iodine solution was used as an internal control standard to evaluate the antibacterial effect of TEO during the time-kill assay.

### 2.6. Preliminary Test for Standardization of In Vitro Synthesis of Biofilm Assay

All ATCC (*S. aureus* 25923 and 11623 and *S. epidermidis* 12228) were inoculated in BHI broth and incubated overnight at 37 °C. Then, these fresh ATCC cultures were cultured on TSA plates overnight at 37 °C. The colonies from overnight, grown on Trypticase Soy Agar (TSA) (Liofilchem, Teramo, Italy) culture plates, were suspended directly into physiological saline (0.89% NaCl) and vortexed to achieve a suspension of 0.5 McFarland turbidity (1.5 × 10^8^ CFU/mL). Each well of sterile 96-well flat-bottom polystyrene tissue culture-treated plates (Sigma Aldrich, Costar, Saint Louis, MO, USA) were filled with a 190 μL aliquot of BHI added with 1% glucose and then 10 μL of bacterial suspension. All experiments were performed in triplicate and incubated overnight (24 h) at 37 °C.

After incubation, the plates were inverted and gently tapped to remove residual broth. The wells were washed four times with 0.2 mL of phosphate buffer saline (PBS) (Merck KGaA, Darmstadt, Germany) (pH 7.2) to remove planktonic bacteria before fixation.

The fixation of cells in the plates was performed by adding 150 μL of methanol for 20 min. After fixation, the well walls of the microplate were stained with 150 μL safranine for 15 min, after which the excess crystal violet was removed and washed twice with PBS until runoff was clear. The plates were left to air-dry for 30 min. The elution was performed with 150 μL of methanol and left at room temperature for 30 min. The elute was read with optical density (OD) readings at 570 nm in an ELISA plate reader (Molecular Devices, San Jose, CA, USA) (Figure S1).

The categorization of isolates according to biofilm formation capacity was performed with the method described below and used for biofilm gradation in clinical isolates.

ODc: optical density control represents the optical density net of the white blank value (empty well = 0.063).

ODs: optical density screening.

OD (ODs-ODc): optical density of each well, which was interpreted as follows:

1. Nonbiofilm producers (OD ≤ ODc);
2. Weak biofilm producers (ODc < OD ≤ 2 × ODc);
3. Moderate biofilm producers (2 × ODc < OD ≤ 4 × ODc);
4. Strong biofilm producers (4 × ODc < OD).

*S. epidermidis* ATCC 12228 served as the negative control. However, *S. aureus* ATCC 25923 (high slime producer) and *S. aureus* ATCC 11632 (moderate slime producer) were used as positive control.

The cutoff value (ODc) can provide the categorization of isolates whether a biofilm producer or not, according to Stepanović and collaborators’ recommendations for assessment of biofilm production by staphylococci [41]. The preliminary development of this cutoff was essential for the purpose of this study to calculate the MBIC, defined as the lowest concentration of an antimicrobial agent required to inhibit the formation of biofilms, which was determined by observing ODc values (OD ≤ ODc = inhibition of biofilm producer). It should also be noted that OD values were averaged and SD was calculated.

### 2.7. Biofilm Production by Isolated Bacteria and TEO Inhibition

Biofilm production was evaluated before and after treatment with TEO solutions at different concentrations (0.25%, 0.5%, and 1.0% *v*/*v*), considering the gold-standard method for biofilm detection described by Mathur et al. [41], detection of biofilm by Microtiter Plate Method (MTP), and the effect of TEO to inhibit biofilm formation was measured using minimum biofilm inhibitory concentration (MBIC). The assay for detection was modified as reported. Organisms isolated from fresh agar plates were inoculated in 10 mL of BHI added with 1% glucose and incubated at 37 °C for 24 h [42]. For each test, individual wells of sterile 96-well flat-bottom polystyrene tissue culture-treated plates (Sigma Aldrich, Costar, Saint Louis, MO, USA) were filled with 200 µL of bacterial suspension. In the same 96-well plates, positive and negative control cultures were added, and sterile broth was used to set up the presence of any possible contamination. The trial was carried out in triplicate. All plates were incubated at 37 °C for 24 h, and after incubation, the contents of each well were removed by gentle tapping. The wells were washed four times with 0.2 mL of phosphate buffer saline (PBS), pH 7.2, to remove free-floating bacteria. Before staining with safranine, fixation of biofilms was carried out by adding 150 μL of methanol for 20 min. Then, the sessile isolates that generated biofilms on the well walls of the microplate were stained with 150 µL safranine for 15 min and washed twice with PBS to discharge the safranine stain. After the air-drying process, the dye of biofilms that coated the microplate walls was resolubilized by 150 µL of methanol. The optical density (OD) of each well was measured using a microplate ELISA reader (Emax, USA) at 570 nm [43]. The cutoff OD (ODc) is defined as three standard deviations above the mean OD of the negative control. Uninoculated wells containing sterile MHB supplemented with 1% glucose were used as blanks. Inoculated wells containing ATCC 25923 and ATCC 11632 were considered positive controls and with ATCC 12228 negative controls.

Isolates with an OD value higher than the blanks were biofilm producers. The cutoff value (ODc) can provide the categorization of isolates whether they are a biofilm producer or not [43]. The MBIC, defined as the lowest concentration of an antimicrobial agent required to inhibit the formation of biofilms, was determined by observing ODc values (OD ≤ ODc = inhibition biofilm producer).

All experiments were performed in triplicate, and the percentage inhibition was calculated as follows:

Inhibition percentage = (OD negative control − OD treated sample/OD negative control) × 100%.

### 2.8. PCR for the Detection of Genes Linked to Biofilm Production

Genomic DNA isolated from a single colony of each sample was extracted using a DNeasy Blood & Tissue Kit (Qiagen, Hilden, Germany), according to the manufacturer’s protocol. The DNA concentration was estimated by a Qubit Fluorometer using a Qubit dsDNA HS Assay (Thermo Fisher Scientific, Waltam, MA, USA). The eluted DNA samples with positive and negative controls for *icaA* and *icaD* (ATCC 25923 and ATCC 12228) were used as a template for the polymerase chain reaction (PCR) method. Two pairs of primers were used for the amplification of the *icaA* and *icaD* genes [44]. For the detection of *icaA*, the following primers were used: 5′-TCTCTTGCAGGAGCAATCAA as the forward primer and 5′-TCAGGCACTAACATCCAGCA as the reverse primer, yielding a PCR product of 188 bp. For the detection of *icaD*, the following primers were used: 5′-TGGTCAAGCCCAGACAGAG and 5′-CGTGTTTTCAACATTTAATGCAA as forward and reverse primers, respectively, yielding a PCR product of 198 bp. PCR amplifications were performed with a 25 µL reaction mixture consisting of 3 µL of genomic DNA, 2.5 µL of 10XPCR Buffer, 0.25 µL of Platinum™ Taq DNA Polymerase (5U/µL) (Invitrogen™, San Giuliano Milanese, Italy), 10 mM dNTPs mix, and 0.5 µL at 10 µM of each primer. To perform the reactions, a Mastercycler EP Gradient thermocycler (Eppendorf, Hamburg, Germany) was used, with the following amplification condition: an initial denaturation step at 94 °C for 2 min, followed by 35 cycles of denaturation (94 °C for 15 s), annealing (55.5 °C for 30 s), and extension (68 °C for 30 s), and the final extension at 68 °C for 5 min. PCR products were analyzed using capillary electrophoresis technology by a QIAxcel Advanced system with a QIAxcel DNA Screening Kit (Thermo Fisher, Italy).

### 2.9. Statistical Analysis

The Shapiro–Wilk test was used to assess the normality of the distribution. For independent samples, a One-way Analysis of Variance (ANOVA) or equivalent nonparametric test was used, followed by Dunnet’s test as a post hoc analysis. A significance level of *p* < 0.05 was chosen to determine statistical significance. Moreover, the possible correlation of TEO activity and the resistance profile of the isolates was investigated (susceptible, resistant to 1 antimicrobial class, and resistant to ≥2 antimicrobial classes). The statistical analyses were performed using GraphPad Prism v8.1.2 (Dotmatics, Boston, MA, USA).

## 3. Results

### 3.1. Chemical Composition of the TEO

The chemical composition TEO was determined by GC/MS. About twenty-five components of TEO were identified, comprising 98.7% of the total detected constituents as previously reported [45]. The major components were thymol (47.01%), o-cymene (19.64%), and γ-terpinene (8.83%), suggesting that TEO belongs to the thymol chemotype (Table S1).

### 3.2. Isolated Bacteria

A total of 12 bacteria from subclinical mastitis were isolated and used in this study. *Staphylococcus* spp. strains were divided into six coagulase-negative staphylococci (CNS), consisting of one *S. xylosus*, two *S. chromogenes* (a,b), one *S. equorum*, two *S. epidermidis* (a,b), and six coagulase-positive staphylococci consisting of *S. aureus* (a–f).

### 3.3. MDR Activity

In total, three of the *Staphylococcus* coagulase-negative isolates were identified as multidrug-resistant (MDR) strains, while two *S. aureus* isolates were identified as methicillin-resistant (MRSA) by the disk diffusion method (Table 1).

### 3.4. Screening for Antibacterial Activity of TEO

#### 3.4.1. Disk Diffusion Assay

The averages of the inhibition diameters were calculated and are reported in Table 2a,b. All tested strains were sensitive to 0.25% (*v*/*v*) and more sensitive at higher concentrations. However, *S. aureus* (f) was resistant to 0.25% (*v*/*v*) concentration, while *S. xylosus* and *S. aureus* (d) showed moderate sensitivity.

#### 3.4.2. MIC and MBC

The bactericidal and/or bacteriostatic properties of TEO were evaluated by MIC and MBC. The MIC value of TEO was obtained at a concentration of 0.50% (*v*/*v*) for a single coagulase-positive isolate strain (*S. aureus* (f)) and at a concentration of 0.25% (*v*/*v*) for all other isolated strains. TEO showed effective bactericidal action with a 99.9% reduction in CFU/mL of all isolated strains in the MBC assay at a concentration of 0.25% (*v*/*v*) for most strains tested, while for *S. epidermidis* (b) and *S. aureus* (e,f), the values of MBC were 0.50% (*v*/*v*) (Table 3).

#### 3.4.3. *Time-Kill Kinetics*

**Figure 1:**
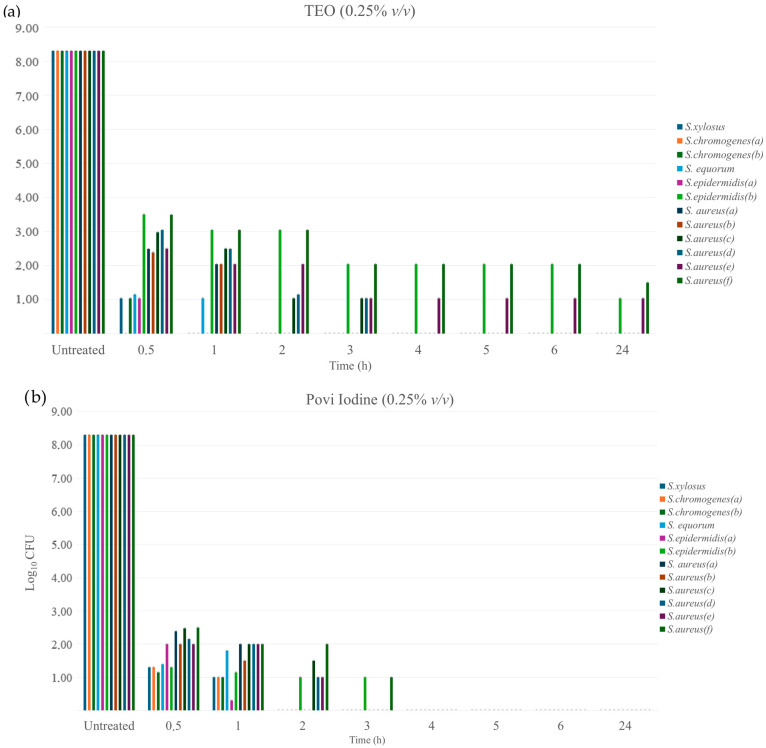
Time-kill of *Staphylococcus* isolates at different times (0–24 h): (**a**) TEO time-kill, both treated with dose of 0.25% (*v*/*v*); (**b**) Povi Iodine time-kill.

The lowest concentration of TEO, which showed the most effective antibacterial activity in broth microdilution tests, was selected for the evaluation of the time-kill kinetics (Figure 1a,b). TEO reduced the number of viable coagulase-negative *Staphylococcus* by 10^7^ CFU/mL (*p* < 0.0001), 10^5^ CFU/mL for *S. aureus* (d,f), and 10^6^ CFU/mL for *S. aureus* (a,b,c,e), isolated after 0.5 h of contact. Moreover, a reduction of 10^7^ CFU/mL was observed after 3 h for most strains except for *S. epidermidis* (b) and *S. aureus* (e,f), in which there was a reduction of 10^6^ CFU/mL (*p* < 0.0001). For most strains, TEO at 0.25% (*v*/*v*) was effective in determining the significant results for all bacteria tested at the end of 3 h contact (*p* < 0.0001), showing complete inhibition effects for 9 out of 12 strains, similar to the results obtained with those of the iodine solution. In contrast, for *S. epidermidis* (b) and *S. aureus* (e,f), the iodine solution at the chosen concentration was able to inhibit growth below limit of detection (*p* < 0.0001).

#### 3.4.4. Production of Biofilm

**Figure 2:**
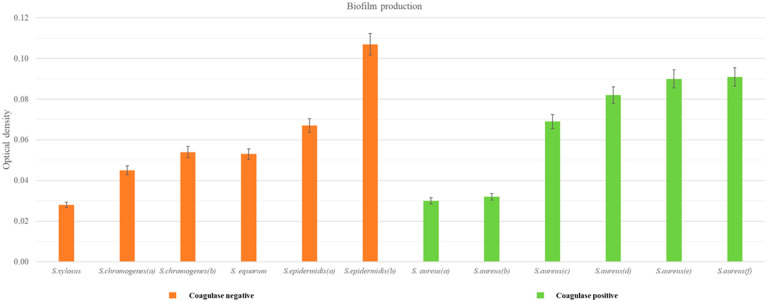
Biofilm production CNS and CPS (*S. aureus*) measured by MTP method.

As shown in Figure 2 and Table 4, a 50% share of the isolates were found to be biofilm-producing by the MTP method. Within the biofilm-positive *S. aureus* strains, 50% of the isolates were found to be medium biofilm producers, whereas among the CNS isolates, only one strain was a medium biofilm producer. In both groups, one strain was identified as a weak biofilm producer.

**Figure 3:**
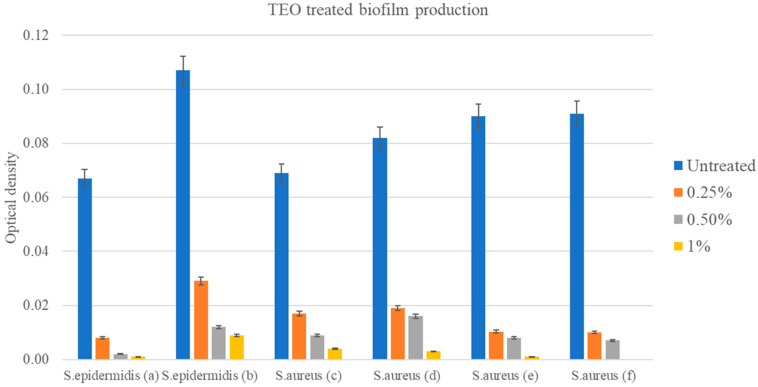
Biofilm productions levels of *S. epidermidis* (a,b) and *S. aureus* (c–f) (strong producers) when treated with TEO solutions at various concentrations (0.25%, 0.5%, and 1.0% (*v*/*v*)) compared with untreated control. Optical density (ODs-ODc) ≤ 0.063 identifies TEO’s ability to inhibit biofilm.

After exposure to TEO at concentrations of 0.25%, 0.5%, and 1.0% (*v*/*v*), significant alterations in biofilm production of medium biofilm producers were observed. Indeed, the application of TEO solutions led to marked inhibition in biofilm formation for both *S. epidermidis* (a,b) and *S. aureus* (c–f) (Figure 3) at all the tested concentrations, recording an OD ≤ ODc (OD ≤ 0.063), with an MBIC value of 0.25%. Statistical analyses conducted on the OD results referring to each bacterial strain revealed significant differences in biofilm production among the various concentrations of TEO when compared with untreated controls (*p* < 0.001).

#### 3.4.5. *Biofilm-Producing Genes*

The PCR technique was applied to 12 DNA samples isolated from staphylococcal strains and the 2 reference ATCC strains, 2 positive controls, and 1 negative control. The slime-producing reference strains ATCC 25923 and ATCC 11632 were found to be positive for both genes, giving a 188 bp band for the *icaA* gene and a 198 bp band for the *icaD* gene. The nonslime-producing *S. aureus* reference strain ATCC 11622 was negative for both genes. Molecular study of *icaA* and *icaD* genes among 12 strains revealed that 5 samples (41.7%), such as *S. aureus* (a), *S. aureus* (b), *S. aureus* (d), *S. aureus* (e), and *S. aureus* (f), which were positive for *icaA* were also positive for *icaD*, while 1 of them, *S. aureus* (c), tested positive only for the *icaD* gene. The remaining isolates (58.3%) did not carry either of these two genes, as reported in Table 5.

#### 3.4.6. TEO Activity and Resistance Profile

Finally, the possible relationship between TEO activity and the resistance profile of isolates was investigated (sensitive, resistant to an antimicrobial class, and resistant to ≥2 antimicrobial classes). Statistical analysis showed no significant differences in TEO activity between the different resistance groups (*p* = 0.706).

## 4. Discussion and Conclusions

During milking, several biosecurity measures must be adopted to minimize the transmission of pathogens as well as the risks for consumers [2]. *Staphylococcus* spp. is often associated with biofilm generation, resulting in firm adhesion and fixation of bacteria on surfaces. Since the three-dimensional polymeric structure of the biofilms prevents the penetration of antimicrobials, controlling biofilm formation could pose a challenge to the food industry and public health worldwide [46].

In farms, nipple disinfection is usually performed using iodine solution, which has been identified as a factor that increases the concentration of iodine in milk, both by direct contamination and by absorption by the epithelium of the nipples [18,19]. Such concentrations of iodine in milk, even if within the legislative limits, may pose a risk to sensitive consumers or sufferers of thyroid disorders [18,19,47].

In this context, the aim of this study was to evaluate the effectiveness of natural products such as EO against *Staphylococcus* spp. isolated mastitogenic agents [48] as an alternative to the use of iodine for nipple disinfection. TEO has demonstrated significant antibacterial efficacy in vitro, proving to be a viable and safer alternative to disinfectants commonly used in milking parlors. In fact, TEO proved effective at the lowest concentration tested, 0.25% (*v*/*v*), against most of the strains tested, both in broth and plates. For *S. aureus* (f), it should be noted that the MIC was higher than for the other strains (0.5%). However, the lower sensitivity of *S. epidermidis* (f) and of the two strains of *S. aureus* (e,f) with a higher MBC value 0.50% (*v*/*v*) than the other staphylococcal strains should be highlighted. The data obtained were largely traced with a disk diffusion assay except for *S. xylosus* and *S. aureus* (d). The MIC, MBC, and disk diffusion values obtained were higher than those reported in other studies [23,49]. However, it should be considered that the different quantitative composition of an EO due to extraction method, harvest season, geographical distribution, and synergistic effects between the various components may contribute to influence its antimicrobial properties [50]. At the same time, it is difficult to define a standard of reference for experimental procedures for assessing the effectiveness of an EO [51].

Another noteworthy aspect is the possible relationship between TEO activity and the resistance profile of the isolates (susceptible, resistant to one antimicrobial class, and resistant to ≥2 antimicrobial classes). However, no statistically significant differences in TEO activity were shown in this study between the different resistance groups.

It is important to emphasize that the present study must be interpreted in the context of its limitations. Among these, the low number of strains tested and the limited array of antibiotics molecules available are the most evident. Indeed, the ability of EOs to inhibit the growth of Gram-positive and Gram-negative bacteria causing bovine mastitis and that are multiresistant to antibiotics is reported in the literature [52]. Further studies are therefore needed to investigate the potential of TEO in combating and preventing infections caused by multidrug-resistant mastitogenic bacteria.

The broad-spectrum activity of TEO can be justified by its chemical composition, thymol (47%), followed by p-cymene (19.6%) and γ-terpinene (9%). It is documented that thymol acts on the integrity of the bacterial cell membrane, promoting the release of potassium ions and intracellular nucleic acids, leading to irreversible damage until cell death [53]. In addition, the p-cymene assists by increasing the action of thymol, contributing to the swelling of the bacterial cytoplasmic membrane and the entry of compounds into the cell [31,54]. Therefore, the choice to use TEO, and not its individual components, arises from preclinical studies present in the literature, which demonstrated that p-cymene, if used alone, does not show an appreciable antibacterial effect even though it prevents biofilm formation [55].

Compliance with hygiene and sanitation standards is a key requirement for the dairy industries, since biofilm formation, associated with insufficient disinfection of the udder or milking parlor, is a critical issue [17,56]. Indeed, bacterial cells can detach from the matrix and contaminate food, leading to premature spoilage of the products and health risks for consumers [57]. Pre- and postdisinfections are essential to reduce nipple contamination before and after milking as well as biofilm formation on the nipples and on the teat cup liners of the automatic milking system [17,56]. Monitoring the biofilm forming ability of *staphylococci* causing mastitis and the genes involved in it may provide new ideas or strategies for the prevention or the effective treatment of bovine mastitis. Consequently, antimicrobial products, such as iodine, chlorine, chlorhexidine, or quaternary ammonia, are widely used to prevent mastitis, and the efficiency of these disinfectants is a critical point in the control of the disease and for success in the dairy activity [58]. However, the use of these disinfectants can leave residues in the milk, which in turn can have dangerous effects on the health of consumers [18,47].

The present study demonstrated that 96% of the isolated strains were potential biofilm producers, carrying the *icaA* and *icaD* genes, but only 50% of them expressed it phenotypically. Among the *ica* genes, it was reported that the *icaA* and *icaD* genes play a significant role in biofilm formation within the genus *Staphylococcus* and particularly in *S. aureus* and *S. epidermidis* [6,59,60]. In fact, it was reported that the expression of *icaA* alone induces low enzymatic activity, while the co-expression of *icaA* and *icaD* leads to a significant increase in enzymatic activity and substantial mucus production [44,61]. In fact, the co-expression of *icaA* and *icaD* is necessary for PIA synthesis, resulting in increased N-acetyl-glucosamine transferase activity and mucus production [30]. An interesting result observed is an isolate strain of *S. aureus* (c) not carrying *icaA* genes that was still able to produce biofilm. This was previously reported by Omidi et al. [62], who identified four MRSA (methicillin-resistant *S. aureus*) strains not carrying *icaA* genes as strong biofilm producers by phenotypic methods. This may indicate that genes other than *ica* may be involved in biofilm formation in these strains [61]. The presence of *icaA*- or *icaD*-negative biofilm-positive isolates can be accounted for by an *ica*-gene-independent control of slime production/adhesion mechanism [30]. Conversely, the inability of *Staphylococcus* isolates positive for *icaA* and/or *icaD* genes to produce biofilm in vitro may be due to point mutation in the locus and/or other yet unidentified factors that negatively regulate the synthesis of intercellular adhesion polysaccharide or influence biofilm formation [11]. Also, some experimental evidence supports the development of new clones referred to as biofilm-negative that are both *icaA*- and *icaD*-gene-positive [44]. However, the biofilm formation of the strains after treatment with TEO were negative for all concentrations tested, reporting an MBIC value of 0.25%. The difference between MBC (0.50%) and MBIC (0.25%) values is noteworthy for some bacterial strains, such as *S. epidermidis* (b) and *S. aureus* (e,f). For those strains, TEO showed greater effectiveness in inhibiting biofilm than in antimicrobial effects. Similar results are reported in the literature [63], explained by the different inhibitory pathways targeted by EOs. Indeed, among the biofilm inhibitory effects associated with this type of natural product, the inhibition of quorum sensing stands out, which in turn decreases the expression of virulence factors and bacterial adhesion to different surfaces [64]; the production of adhesins and exopolysaccharides [65]; proton motor force collapse due to ATPase inhibitory activity; energy decrease in the form of ATP; and substance flow blockage [66]. Therefore, it is more useful to define both the MIC and MBIC values, as opposed to the MIC value alone, in order to formulate two different solutions at different concentrations of TEO: (i) pre- and postdipping on the teat to minimize the potential irritating effect on the skin during topical use, and (ii) pre- and postmilking for optimization of costs, with bactericidal disinfectant activity (higher) and antibiofilm (lower) concentrations.

The efficacy of various plant extracts in preventing biofilm formation in various microorganisms, including *S. aureus*, has been repeatedly demonstrated [20,22,25]. The results of the present study showed that TEO has an excellent effect on preventing biofilm formation, in agreement with previous findings in the literature [25,67].

Considering the volatile nature of some components of the essential oil and its photosensitivity, which could reduce the efficacy and contact time with treated surfaces, it was important and useful to know and evaluate the action time of TEO, a mark of TEO’s antibacterial power, during total contact of 24–48 h through time-kill. In this assay, the kinetics of the decrease in the number of viable cells was evaluated (from 30 min to 24 h) to the tested dilution of 0.25%. This value coincides with the MIC of TEO for most strains tested. This evaluation, which was also performed at the same concentration for the iodine solution, showed that Povi-Iodine and TEO are able to significantly reduce initial bacterial concentrations in a similar way 3 h after the start of contact. In particular, the iodine solution appears to be more effective against biofilm-producing bacteria at the tested concentration. However, despite the fact that there was no complete inhibition of microbial growth for *S. epidermidis* (f) and *S. aureus* (e,f) strains, it should be noted that these strains had a higher MIC than the tested dilution value, and after 3 h of contact, the starting bacterial concentration was significantly reduced from ~1 × 10^8^ to ~1 × 10^2^ CFU/mL (*p* < 0.0001). Thus, these results confirm that the action of TEO may depend on the contact time, concentration used, and strain being treated. Our findings are corroborated by a recent study by Naccari et al. (2019), which highlighted the efficacy of TEO against multiresistant *S. aureus* and *S. epidermidis* responsible for nodular dermatitis aggravated by mastitis symptoms in sheep [68]. Considering this, it is important to emphasize that the use of EOs protects consumers from the presence of iodine residues in milk without causing sensory changes, such as undesirable smell or taste of milk or dairy products, due to their low dosage and the liability of their permanence on udder tissue. Therefore, it is desirable to conduct further research to better evaluate the effect of TEO on udder health and consumer protection.

Considering other studies evaluating the activity of EOs on bacteria responsible for mastitis [20], this is the first report on the activity of EOs, in particular TEO, which evaluates the antibacterial efficacy against mastitis-causing *Staphylococcus* spp., also considering time-kill analysis. Based on the broad-spectrum antibacterial potential of EOs, we can assert that pre- or postdipping (of lactating dairy animals with TEO with a suggested concentration from 0.25% to 0.50% (*v/v*), corresponding to 2.32–4.64 mg/mL (*w*/*v*)) is a promising alternative for microbial control and sanitization of the nipple surface, allowing to reduce income losses and providing consumers with a safer product. Unlike disinfection of the teat-holder sleeves of milking machines, TEO formulations at a concentration of 0.25% (2.32 mg/mL (*w*/*v*)) appears to be sufficient to prevent the formation of biofilm and with it the colonization and spread of mastitogenic agents within the entire herd. The data obtained, as already mentioned, do not align completely with those present in the literature due to various variability factors (composition of the EO, lack of standardization of the method, variability of the strain under examination, etc.). However, Gupta et al. [69] report an efficacy range of TEO equal to 0.8 mg/mL–6.25 mg/mL, while the range for the diffusion test in plates stands at values of 9–12 mm/5 µL.

In conclusion, considering the risk factors for the consumer due to iodine residues in milk and the known effect of the widespread and indiscriminate use of biocides that contribute to the selection and proliferation of existing bacteria [70,71], it seems more urgent than ever and necessary to shift attention to natural substances whose residues in milk are not a concern and which exhibit antimicrobial and antibiofilm activity while counteracting the emergence of antibiotic resistance, as reported by Bouyahya et al. [72]. In light of this and our promising results, corroborated by data obtained from similar studies in the literature, we lay the groundwork for further investigation to assess the stability of the product for the formulation of essential-oil-based products in liquid, gel, foam, or spray form for topical use on skin or equipment. For this reason, our research promises new steps to be implemented in the near future, such as testing of cytotoxicity in vitro, and then, in collaboration with the chemistry section, to start in vivo tests with dosages and formulations useful for the production of commercial products with low costs in order to realize the objectives of our study: reducing antibiotic consumption, safeguarding animal and consumer health, and improving parlor hygiene. However, right now, this study provides new data and complements the existing literature from One Health and Green Veterinary Pharmacology perspectives.

## Figures and Tables

**Table 1:** (a,b) Antibiotic susceptibility of the *Staphylococcus* isolates.

(a)						
Antibiotics	*Staphylococcus* Coagulase-Negative					
	*S. xylosus*	*S. chromogenes* (a)	*S. chromogenes* (b)	*S. equorum*	*S. epidermidis* (a)	*S. epidermidis* (b)
AML	R	S	S	S	R	R
AMP	I	I	I	I	R	R
P	I	I	R	I	R	R
CL	I	S	I	S	I	I
FOX	I	S	S	S	I	I
CRO	S	S	S	S	S	S
CD	R	S	S	S	S	R
TE	R	S	S	S	S	R
OX	I	S	S	S	R	R
CN	S	S	S	S	I	I
E	R	S	I	S	S	R
RD	S	S	S	S	I	R
ENR	S	S	S	S	S	S
**(b)**						
**Antibiotics**	***Staphylococcus*** **Coagulase-Positive**					
	***S. aureus***	***S. aureus***	***S. aureus***	***S. aureus***	***S. aureus***	***S. aureus***
AML	R	R	R	I	I	R
AMP	R	R	R	I	S	R
P	R	R	R	I	S	R
CL	I	S	R	S	S	R
FOX	I	S	R	S	I	R
CRO	S	S	I	S	S	I
CD	S	S	I	S	S	S
TE	S	S	R	S	S	R
OX	S	S	R	S	S	R
CN	S	S	S	S	S	S
E	R	R	R	S	S	R
RD	S	S	I	S	S	R
ENR	S	S	S	S	S	S

Antibiotics: amoxicillin (AML; 30 μg), ampicillin (AMP; 10 μg), penicillin (P; 10 μg), cefalexin (CL; 30 μg), cefoxitin (FOX; 30 μg), ceftriaxone (CRO; 30 μg), clindamycin (CD; 2 µg), tetracycline (TE; 30 μg), oxacillin (OX; 1 μg), gentamicin (CN; 10 μg), erythromycin (E;15 µg), rifampicin (RD; 30 μg), enrofloxacin (ENR; 5 μg).

**Table 2:** TEO antimicrobial sensitivity tests of isolate strains by disk diffusion method.

(a)						
*Staphylococcus* Coagulase-Negative						
%TEO (*v*/*v*)	*S. xylosus*	*S. chromogenes* (a)	*S. chromogenes* (b)	*S. equorum*	*S. epidermidis* (a)	*S. epidermidis* (b)
1%	+++	+++	+++	+++	+++	+++
0.5%	+++	++	+++	+++	+++	++
0.25%	+	++	++	++	++	+
**(b)**						
***Staphylococcus*** **Coagulase-Positive**						
**%TEO (*v/v*)**	***S. aureus*** **(a)**	***S. aureus*** **(b)**	***S. aureus*** **(c)**	***S. aureus*** **(d)**	***S. aureus*** **(e)**	***S. aureus*** **(f)**
1%	+++	+++	+++	+++	+++	+++
0.5%	+++	+++	++	++	+++	++
0.25%	++	++	++	+	++	-

The tables show the averages of inhibition diameters expressed as follows: (i) less than or equal to 8 mm = resistant (n.i.); (ii) 8–14 mm = moderately sensitive (+); (iii) 14–20 mm = sensitive (++); (iv) greater than 20 = very sensitive (+++).

**Table 3:** MIC and MBC values of TEO (%) tested against the bacterial strains.

Coagulase-Negative Strains	MIC	MBC
*S. xylosus*	0.25%	0.25%
*S. chromogenes* _(a)_	0.25%	0.25%
*S. chromogenes* _(b)_	0.25%	0.25%
*S. equorum*	0.25%	0.25%
*S. epidermidis* _(a)_	0.25%	0.25%
*S. epidermidis* _(b)_	0.25%	0.50%
**Coagulase-Positive Strains**	**MIC**	**MBC**
*S. aureus* _(a)_	0.25%	0.25%
*S. aureus* _(b)_	0.25%	0.25%
*S. aureus* _(c)_	0.25%	0.25%
*S. aureus* _(d)_	0.25%	0.25%
*S. aureus* _(e)_	0.25%	0.50%
*S. aureus* _(f)_	0.50%	0.50%

**Table 4:** Biofilm production CNS and CPS (*S. aureus*) measured by optical density (OD).

Coagulase-Negative Strains	ODs *	OD (ODs-ODc) **
*S. xylosus*	0.087	0.028
*S. chromogenes* (a)	0.104	0.045
*S. chromogenes* (b)	0.113	0.054
*S. equorum*	0.115	0.053
*S. epidermidis* (a)	0.130	0.067
*S. epidermidis* (b)	0.166	0.107
**Coagulase-Positive Strains**	ODs *	OD (ODs-ODCc *) **
*S. aureus* (a)	0.093	0.030
*S. aureus* (b)	0.095	0.032
*S. aureus* (c)	0.132	0.069
*S. aureus* (d)	0.145	0.082
*S. aureus* (e)	0.153	0.090
*S. aureus* (f)	0.154	0.091

* ODc: optical density control represents the optical density net of the white blank value (empty well = 0.063). * ODs: optical density screening. ** OD (ODs-ODc): optical density of each well, which was interpreted as follows: (1) nonbiofilm producers (OD ≤ 0.063); (2) weak biofilm producers (≤0.063 < OD ≤ 0.126); (3) moderate biofilm producers (0.126 < OD ≤ 0.252); (4) strong biofilm producers (0.252 < OD).

**Table 5:** Results of molecular study on staphylococcal strains to detect icaA and icaD genes.

Coagulase-Negative Strains	icaA	icaB
*S. xylosus*	-	-
*S. chromogenes* (a)	-	-
*S. chromogenes* (b)	-	-
*S. equorum*	-	-
*S. epidermidis* (a)	-	-
*S. epidermidis* (b)	-	-
**Coagulase-Positive Strains**	**icaA**	**icaB**
*S. aureus* (a)	+	+
*S. aureus* (b)	+	+
*S. aureus* (c)	-	+
*S. aureus* (d)	+	+
*S. aureus* (e)	+	+
*S. aureus* (f)	+	+
**Control Strains**	**icaA**	**icaB**
ATCC 25923	+	+
ATCC 11632	+	+
ATCC 12228	-	-

## Data Availability

The original data presented in this study are included in the article.

## Supplementary Materials

The following supporting information can be downloaded at: https://www.mdpi.com/article/10.3390/pathogens14050412/s1, Table S1: Detailed description of TEO chemotypes. Figure S1: Microtiter plate assay of biofilm formation for sample.
