# Supplementary material for: Inhibition of Biofilm Production and Determination of In Vitro Time-Kill Thymus vulgaris L. Essential Oil (TEO) for the Control of Mastitis in Small Ruminants
Source: Pathogens. 2025 Apr 24;14(5):412. doi: 10.3390/pathogens14050412 (PMC12114205; doi:10.3390/pathogens14050412)
Supplement: Supplementary file 1 [file pathogens-14-00412-s001.zip › Figure S1 Microtitre plate assay of biofilm formation for sample.pdf]

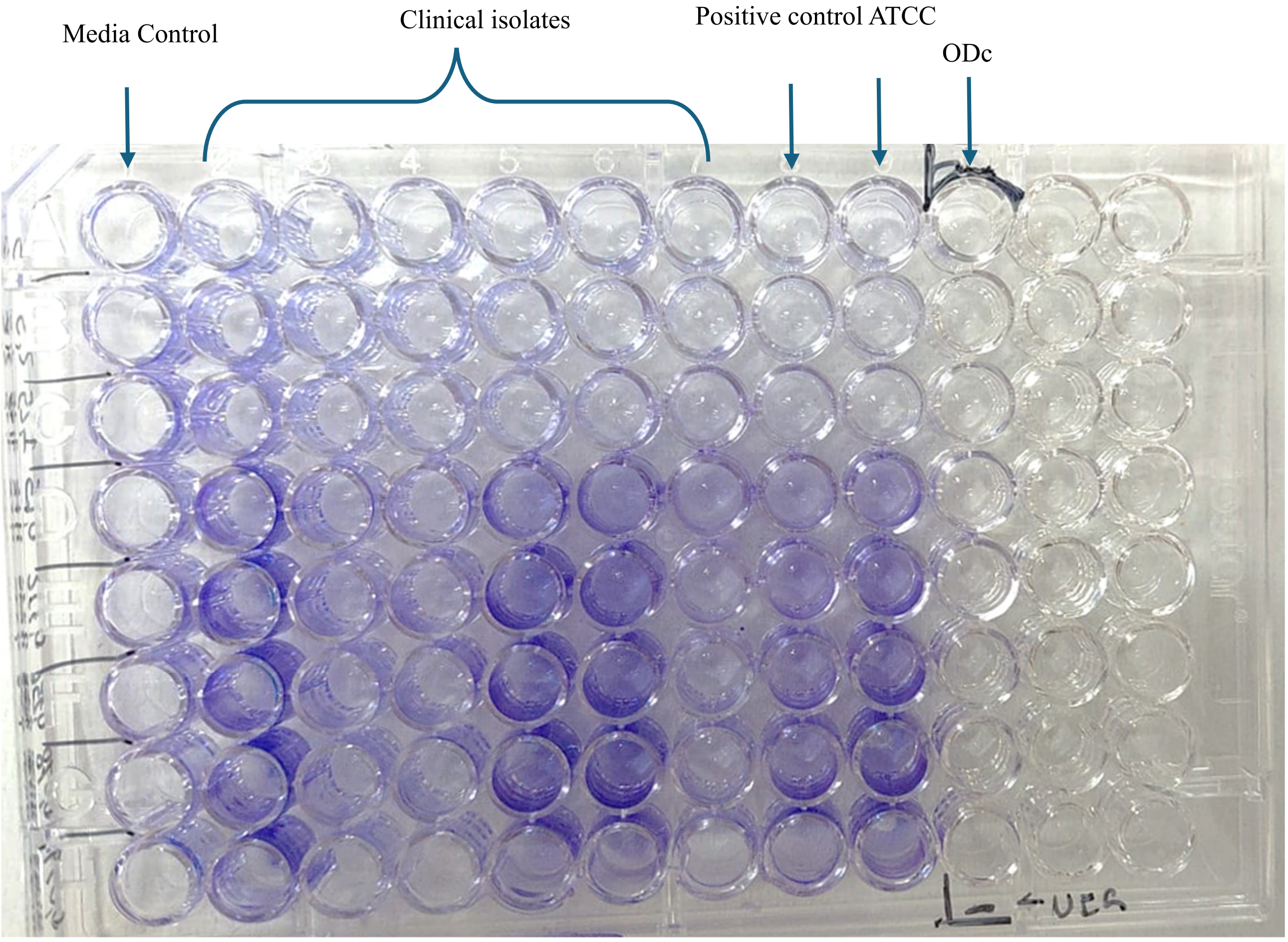

Figure S1. (Supplementary File) - Microtiter plate assay of biofilm formation for sample

Media control: sterile broth to set up the presence of any possible contamination

Clinical isolates: *S. aureus*(c), *S. aureus*(d), *S. aureus*(e), *S. aureus*(f), *S. epidermidis*(b), *S. epidermidis* (a)

Positive control: ATCC: *S. aureus* 11623 and 25923

ODc: optical density control represents the optical density net of the white blank value (empty well= 0.063)
