# Supplementary material for: Inhibition of Biofilm Production and Determination of In Vitro Time-Kill Thymus vulgaris L. Essential Oil (TEO) for the Control of Mastitis in Small Ruminants
Source: Pathogens. 2025 Apr 24;14(5):412. doi: 10.3390/pathogens14050412 (PMC12114205; doi:10.3390/pathogens14050412)
Supplement: Supplementary file 1 [file pathogens-14-00412-s001.zip › Supplementary Table S1.pdf]

**Table S1 -** Detailed description of TEO chemotypes.

| N | Components | AI | TEO |
|---|---|---|---|
| | | | Area±SEM |
| 1 | α-pinene[a] | 931 | 1.81±0.100 |
| 2 | camphene[a] | 952 | 1.89±0.110 |
| 3 | β-thujene | 968 | 0.71±0.060 |
| 4 | β-pinene[a] | 980 | 0.56±0.030 |
| 5 | α-phellandrene[a] | 1003 | 0.15±0.010 |
| 6 | 3-carene[a] | 1016 | |
| 7 | o-cymene | 1021 | 19.64±1.500 |
| 8 | eucalyptol[a] | 1023 | 0.89±0.050 |
| 9 | limonene[a] | 1032 | 0.60±0.040 |
| 10 | γ-terpinene[a] | 1064 | 8.83±1.030 |
| 11 | β-linalool[a] | 1101 | 4.07±1.020 |
| 12 | endo-borneol[a] | 1167 | 1.85±0.700 |
| 13 | estragole[a] | 1198 | |
| 14 | citral[a] | 1240 | |
| 15 | geraniol | 1254 | |
| 16 | anethole[a] | 1284 | |
| 17 | bornylacetate[a] | 1289 | |
| 18 | thymol[a] | 1290 | 47.01±3.320 |
| 19 | geranylaceate | 1385 | |
| 20 | caryophyllene[a] | 1415 | 2.18±0.870 |
| 21 | α-bergamotene | 1430 | |
| 22 | humulene | 1451 | |
| 23 | α-curcumene [a] | 1481 | |
| 24 | zingiberene [a] | 1493 | |
| 25 | β-sesquiphellandrene [a] | 1521 | |
| 26 | caryophylleneoxyde | 1592 | 0.58±0.030 |

[a]: Standard compounds. Arithmetic index (AI) was taken from Adams [22, 23] and/or the NIST 2017 Database. Relative percentage values are a means of three determinations with a structural equation modeling (SEM) in all cases below 10%.
